# Avian influenza A/H7N9 risk perception, information trust and adoption of protective behaviours among poultry farmers in Jiangsu Province, China

**DOI:** 10.1186/s12889-017-4364-y

**Published:** 2017-05-18

**Authors:** Bin Cui, Qiuyan Liao, Wendy Wing Tak Lam, Zong Ping Liu, Richard Fielding

**Affiliations:** 1 Business College, Yangzhou University, Jiangsu Province, China; 2 Jiangsu Co-innovation Center for Prevention and Control of Important Animal Infectious Diseases and Zoonoses, Yangzhou, China; 3 Division of Behavioural Sciences, School of Public Health, The University of Hong Kong, 21 Sassoon Road, Pokfulam, Hong Kong, Special Administrative Region China; 4 College of Veterinary medicine, Yangzhou University, Jiangsu Province, People’s Republic of China

**Keywords:** Influenza A (H7N9), Risk perception, Information trust, Behaviour, Poultry farmers

## Abstract

**Background:**

Poultry farmers are at high-risk from avian influenza A/H7N9 infection due to sustained occupational exposures to live poultry. This study examined factors associated with poultry farmers’ adoption of personal protective behaviours (PPBs) based on Protection Motivation Theory (PMT).

**Methods:**

Totally, 297 poultry farmers in three cities of Jiangsu Province, China were interviewed during November 2013-January 2014. Data on PMT constructs, perceived trustworthiness of A/H7N9 information from mass media (formal sources), friends and family (informal sources), intention to adopt and actual adoption of PPBs and respondents’ demographics were collected. Structural equation modeling (SEM) identified associations between demographic factors and PMT constructs associated with A/H7N9-oriented PPB intention. Moderated mediation analysis examined how demographics moderated the effects of information trust on PPB intention via risk perceptions of A/H7N9.

**Results:**

Respondents generally perceived low vulnerability to A/H7N9 infection. The SEM found that male respondents perceived lower severity of (β = −0.23), and lower vulnerability to (β = -0.15) A/H7N9 infection; age was positively associated with both perceived personal vulnerability to (β = 0.21) and perceived self-efficacy (β = 0.24) in controlling A/H7N9; education was positively associated with perceived response efficacy (β = 0.40). Furthermore, perceived vulnerability (β = 0.16), perceived self-efficacy (β = 0.21) and response efficacy (β = 0.67) were positively associated with intention to adopt PPBs against A/H7N9. More trust in informal information (TII) was only significantly associated with greater PPB intention through its positive association with perceived response efficacy. Age significantly moderated the associations of TII with perceived Self-efficacy and perceived response efficacy, with younger farmers who had greater TII perceiving lower self-efficacy but higher response efficacy.

**Conclusion:**

Poultry farmers perceive A/H7N9 as a personally-irrelevant risk. Interventions designed to enhance perceived response efficacy, particularly among lower educated respondents may effectively motivate adoption of PPBs. Informal information may be an important resource for enhancing response efficacy.

**Electronic supplementary material:**

The online version of this article (doi:10.1186/s12889-017-4364-y) contains supplementary material, which is available to authorized users.

## Background

The first human cases of avian influenza A (H7N9) were reported in eastern China in March 2013 [1], subsequently spreading to over 10 provinces in China [2]. Fortunately, the virus transmits inefficiently between humans [3, 4]. Genomic analysis suggests that A/H7N9 virus is of avian origin and mainly transmitted through exposure to infected poultry [1]. The median age of confirmed A/H7N9 human cases was 61 years [3], indicating that older people are a high risk group for A/H7N9 infection. Over 60% of confirmed A/H7N9 human cases reported a history of exposure to live poultry [2]. This raises concerns that those in frequent contact with poultry such as poultry traders and poultry farmers are at high risk of A/H7N9 infection. Although only 6% of confirmed A/H7N9 human cases were poultry workers [3], one previous study reported that over 50% of the surveyed poultry workers had seroconversion for A/H7N9 virus from May 2013 to December 2013 in Southern China though none had virologically confirmed A/H7N9 infection [5]. This indicates that people with occupational exposure to poultry could have a high risk of mild or asymptomatic A/H7N9 infection. More recently, one study indicates that poultry farms could be important sources of reassortment between A/H7N9 virus and other strains of avian influenza viruses [6]. Therefore, poultry farmers may have a high risk of A/H7N9 infection.

The “China Animal Industry Yearbook 2011” reports China having at least 44,061,961 poultry farmers [7]. Large-scale migration in 1990s China saw younger adults migrate from rural to urban areas becoming factory workers [8], sharply raising the mean age of the remaining rural residents with proportions of residents greater than 60 years increasing from 10.9% in 2000 to 15.0% in 2010 [9]. Therefore, many rural Chinese poultry farmers are probably older and potentially more vulnerable to A/H7N9 infection.

Understanding how people at high-risk respond to the outbreak of this novel influenza can guide public health interventions. For example, previous studies identified that an erroneous belief that cooking was the best way of protection from avian influenza could reassure continuing buying of live poultry from wet markets the public [10] and that live poultry traders generally failed to recognize the risks from contact with bird secretions or droppings [11]. All these knowledge deficits could be addressed by public health education to improve protective behaviours. How people perceive the risk of a novel influenza appears to partially influence their protective behaviours [12–14]. However, although the relationships between risk perception and self-protective behaviours have been widely examined in many descriptive studies following novel influenza outbreaks, many are atheoretical [14] and this limits the confidence we have in the veracity of the findings. A theoretical basis is important because it generates testable predictions that build confidence in the validity of the underlying processes. Studies suggest that protective behaviours in response to newly emerging infectious disease outbreaks differ by respondents’ socio-demographic characteristics, particularly age, gender and educational attainment [14] possibly because these variables influence perception of risk [15, 16]. However, few studies have tested these hypotheses within any theoretical frameworks. Studies of pandemic influenza A/H1N1 found that older respondents perceived greater severity of, but lower personal susceptibility to the disease while males generally perceived lower severity of and personal susceptibility to the disease [15, 17]. Few studies reported the relationships between educational attainment and risk perception of influenza. However, higher educational level has been consistently associated with lower perceived risk from other health threats [18, 19], possibly because higher educated people are more likely to be unrealistically optimistic when evaluating their personal risk [19] which might imply greater personal agency or self-efficacy [20]. In relation to experience, farmers with more experience in raising poultry report more familiarity with poultry diseases and thereby perceived lower risk from avian influenza and higher confidence in preventing the disease [13, 21]. Consequently, the first objective of this study was to examine how A/H7N9-related risk perceptions and demographics including age, gender, educational attainment and working experience (indicating by years of raising poultry) influenced intention to adopt personal protective behaviours (PPBs) against A/H7N9. We hypothesized that demographics influence intention to adopt PPBs against A/H7N9 through their effects on A/H7N9-related risk perceptions.

Sources of information are important when considering threat-related information veracity. We distinguish between different information sources as follows: Learning from the experience of the 2003 SARS outbreak in China, the Chinese government actively disseminated information about A/H7N9 through traditional mass media (e.g., TV, radio and newspaper) since it emerged in China in March 2013 [22–24]. Traditional mass media in Mainland China are mainly regarded as government-agency sources for information of infectious diseases and thereby such information is assumed to constitute “formal information” in this study. Information of A/H7N9 disseminated through media is likely to provoke widespread public discussion about the topic. In contrast, information communicated through casual interpersonal communication between friends and family constitutes “informal information” for the purposes of this study. Trust is a core element for effective risk communication, particularly for uncertain infection risks where the risk-related threat is usually invisible [25]. Causal models of trust propose that information trust influences behavioural change indirectly through alterations in risk perceptions [26–28]. The literature on trust suggests two main types of trust can be identified; trust based on judgments of the intentions of others (relational trust) and the trust based on judgments of competence (calculative trust) [29]. While trust in informal information (TII) approximates to relational trust, trust in government (formal) information approximates to calculative trust [29]. Therefore, we propose that trust in formal and informal information may function differentially to motivate behavioural change through their effects on risk perceptions [29]. Previous studies conducted among the general public during the 2009 influenza A/H1N1 pandemic suggests that while trust in formal information was significantly associated with perceived confidence in preventing the disease (efficacy appraisal), TII was significantly associated with perceived risk of the disease (threat appraisal) [30]. Furthermore, the degree of trust in health information from various sources differed by demographics including age, gender and educational attainment [31]. Therefore, it seems plausible that demographics including age, gender and education attainment exert their effects by modifying the effects of information trust on risk perceptions. Therefore, the second objective of the current study was to examine whether demographics including age, gender and educational attainment could modify the indirect effects of information trust on intention to take protective behaviours through risk perceptions related to A/H7N9. Due to lack of available data, no hypotheses about the direction of the moderated effects were set for this objective.

## Methods

### The theoretical framework

**Fig. 1:**
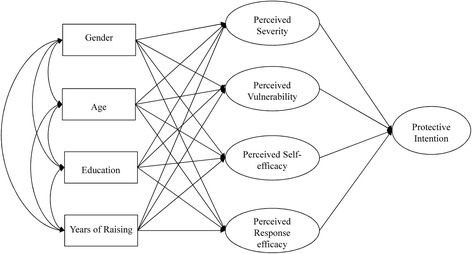
The conceptual framework based on Protection Motivation Theory for understanding farmers’ intention to adopt protective behaviours against avian influenza A/H7N9

This study was designed based on Protection Motivation Theory (PMT) which has been used successfully to predict a variety of behaviours [32, 33]. Many studies have suggested that PMT provides a useful theoretical framework for understanding people’s response to threat-related information during outbreaks of newly-emerging respiratory infectious diseases [14, 33]. PMT focuses on individuals’ cognitive processes in response to fear appeal messages. It proposes that four core cognitive processes mediate the effects of fear appeal messages on motivation to adopt protective behaviours [32]. These four core cognitive processes are perceived Vulnerability (i.e. subjective estimates of the chance of contracting a disease), perceived Severity (i.e., subjective estimates of the seriousness of a disease), perceived Self-efficacy (i.e., the belief that one can successfully take the preventive behaviours) and perceived Response Efficacy (i.e., the belief that existing preventive behaviours are effective in reducing risk of the disease) [34]. PMT also predicts that individual characteristics influence motivation for behavioural change through their effects on these four cognitive components [32]. In this study, we hypothesized that poultry farmers’ demographics, including gender, age, educational attainment and years of raising poultry influence the PMT constructs of perceived Vulnerability (to A/H7N9), perceived Severity (of A/H7N9), perceived Self-efficacy and perceived Response Efficacy in controlling H7N9, which in turn influence poultry farmers’ intention to adopt PPBs against A/H7N9. Figure 1 outlines the conceptual model used. According to the hypotheses of PMT, all the four core components, perceived Vulnerability, perceived Severity, perceived Self-efficacy and perceived Response Efficacy, are hypothesized to be positively associated with intention to adopt PPBs.

Due to limited literature on the relationship between demographics variables and PMT constructs related to avian influenzas, we hypothesized a model comprising saturated relationships (testing all possible relationship permutations) between specified demographic variables and PMT constructs (Fig. 1). Drawing on prior studies we hypothesized that females would perceive higher Vulnerability to and higher Severity of A/H7N9 while older people would perceive lower Vulnerability to but higher Severity of A/H7N9; farmers with more years’ experience of raising poultry would perceive lower Vulnerability to A/H7N9 and lower Severity of A/H7N9 infection, but perceive higher Self-efficacy and Response Efficacy in preventing A/H7N9. For other associations between demographics and PMT constructs, no hypotheses about the directions of associations were set due to lack of prior data.

### Sampling

In the 2013–2014 A/H7N9 outbreak in Mainland China [35], around 52.3% of the cases were reported from Zhejiang, Jiangsu and Shanghai, three provinces located in eastern China along the Yangtze River delta. A total of 59 A/H7N9 human cases had been reported as of December 31, 2014 in Jiangsu Province, with a fatality rate of around 29.6% [36]. Around 28.2% of all confirmed cases of A/H7N9 between March 2013 and June 2014 in China were farmers and around 6% were poultry framers or workers [37]. It was estimated that there were at least 1,094,505 poultry farmers in Jiangsu Province in 2011 [7]. This study was conducted in three cities of Jiangsu Province: Suqian, Nantong and Zhenjiang (Additional file 1: Figure S1).

A/H7N9 virus has been isolated from various birds including pigeons, chickens and ducks [38] but viral shedding is higher and more prolonged in quails and chickens compared to other species [39]. Considering that type of poultry may be a factor that influences poultry farmers’ A/H7N9 risk perceptions and that chicken is the dominant type of poultry raised by these poultry farmers, this study only recruited poultry farmers who raised chickens.

**Fig. 2:**
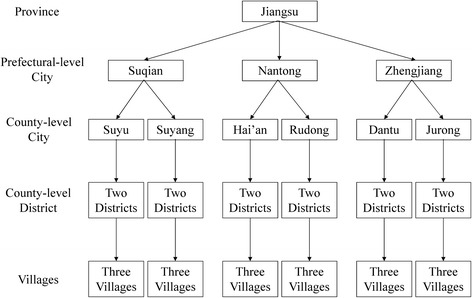
The flow chart showing the process of sampling

Subjects were recruited using a mixed strategy of stratified sampling and random sampling (Fig. 2). Firstly, three prefectural-level cities located in the northern, central and southern parts of Jiangsu Province respectively were selected. Within each selected prefectural-level city, two county-level cities were randomly selected from all those within the prefectural-level city, and within each county-level city, two county-level districts were randomly selected. Following this, three villages were randomly selected from each selected county-level district. Finally, around 10 poultry farmers within each selected village were randomly selected according to the name lists provided by local veterinary authorities (which must record all licensed poultry farms), and approached by the trained researcher for the face-to-face interview. A flow chart showing the process of sampling was provided (Fig. 2).

### Ethics, consent and permissions

This study was conducted during November 2013 to January 2014 following ethical approval from the Yangzhou University and local veterinary bureau which is mainly responsible for distributing avian influenza prevention guideline and monitoring the poultry health and the health of people who work with poultry in Mainland China. The target subjects were first given an explanation of the study and then their consent to participate was sought. Those agreeing completed a face-to-face interview using a standardized questionnaire. The questionnaire was fully anonymous without collecting any personal identity information. Farmers who were not at home at the time when they were approached or refused to participate were replaced with their nearest neighbor poultry farmers, again based on the veterinary authorities’ lists. Each subject who completed the survey was presented with a small gift (a towel and soap).

### Study instrument

A questionnaire was designed to measure major constructs of PMT including perceived Vulnerability to and perceived Severity of A/H7N9, perceived Self-efficacy and perceived Response Efficacy for protecting against A/H7N9 infection, the intention to adopt, and actual adoption of PPBs against A/H7N9 infection, degree of trust in information about A/H7N9 from traditional mass media (formal), family and friends (informal) sources and finally demographics including gender, age, education and the number of years spent raising poultry.

Specifically, the measures for perceived Severity (4 items), perceived Vulnerability (4 items), perceived Self-efficacy (4 items), perceived Response Efficacy (4 items), and protective intention (3 items) were adapted from earlier pre-validated studies [40–42]. For these items, the respondents were asked to indicate on a 7-point Likert-type scale their level of agreement or disagreement with each statement in the questionnaire (ranging from “1 = very strongly disagree” to “7 = very strongly agree”). The measure of actual PPBs included seven questions that asked respondents if they had adopted each of seven protective behaviours (wearing gloves, wearing protective clothes, wearing a face mask, wearing a protective hat, wearing protective shoes, washing hands after touching dead poultry, washing hands after touching poultry feces (Yes/No)) in their routine husbandry practices. Protective behaviour adoption was recorded as “1”. Otherwise, “0” was recorded. These seven protective behaviours are recommended by the National Health and Family Planning Commission of China in their proposal for personal protection against highly pathogenic avian influenza for high risk persons including the poultry workers and farmers, aiming to reduce their risk of contracting avian influenza viral infection due to occupational exposure to poultry [43]. Two items, each assessing trust in information about A/H7N9 from formal (e.g., how much do you trust the information about A/H7N9 influenza from newspaper, TV and radio?) and informal sources (e.g., how much do you trust the information about A/H7N9 influenza from you friends or relatives?), respectively, were measured with a 5-point Likert-type scale (ranging from “1 = do not believe” to “5 = fully believe”). Items for measuring the PMT constructs and basic descriptive data were shown in Additional file 2: Table S1.

The questionnaire was pretested for its comprehensibility and length among 45 chicken farmers from a country of Suqian city in October 2013 before being formally used in the survey. Minor amendments were made for items that were not easily understood by the farmers but the original meanings of the items were retained.

### Data analysis

To assess the reliability and validity of measures for the PMT constructs including perceived Vulnerability to and perceived Severity of A/H7N9 infection, perceived Self-efficacy and Response Efficacy for preventing A/H7N9 and intention to adopt PPBs against A/H7N9, Cronbach’s alpha (α) coefficients for each latent variable were first calculated. All α values exceeded 0.80 (Additional file 2: Table S2), indicating high internal consistency (internal reliability) for the measures [44]. Then, the average variance extracted (AVE) was used to assess the validity of all these scales. A value of AVE greater than 0.5 for a latent variable indicates a good convergent validity for that variable [45]. The results showed that the AVE values of all PMT constructs exceeded 0.80, suggesting high convergent validity for these latent variables. Using the Fornell–Larcker criterion, the square root of all AVE values (the diagonal values in Additional file 2: Table S2) were higher than the correlations between all latent variables (off-diagonal values) indicating that each latent variable shares more variance with its assigned indicators than with any other latent variable. Such results suggest good discriminant validity for each latent variable.

The conceptual model (Fig. 1) was tested using structural equation modelling (SEM) with demographic variables entered into the model as observed covariant variables and PMT constructs entered as latent variables. All covariance, factor loadings, measurement errors, disturbances and path coefficients were estimated using robust maximum likelihood (MLR) estimator [45]. Path coefficients with *p*-values less than 0.05 were considered as statistically significant. Multiple model fit indices including CFI, TLI, RMSEA and SRMR was used to assess the global model fit. Values of CFI and TLI great than 0.9, of RMSEA and SRMR less than 0.8 suggest an acceptable fit of the model to data. The local fit of the model was assessed by investigating the residual matrix. Since the model was run with MLR estimator, the Satorra-Bentler scaled chi-square difference test [46] was used to compare nested models in order to identify the optimal and more parsimonious model. The direct effects of risk perceptions and indirect effects of demographic on Intention to adopt protective behaviours through risk perceptions were calculated using Bootstrapping methods.

To assess whether the effects of information trust on Intention to take protective behaviours through A/H7N9-related risk perceptions could be modified by age, gender and educational attainment, two analytic steps were adopted. First, we tested the simple mediation model which hypothesized that the effect of information trust on Intention to take protective behaviours was mediated by perceived Severity, perceived Vulnerability, perceived Self-efficacy and perceived Response efficacy. Once the simple mediation relationship was established, multiple group modelling was conducted to examine the conditional indirect effect for each moderator (i.e., gender, age and education). Bootstrapping methods were used to calculate the 95% confidence interval of specific conditional indirect effects. Significant difference in conditional indirect effects across stratum of the moderator indicates significant moderated effect on the mediation relationship. All analyses were conducted using Mplus 7.0.

## Results

### The participants

A total of 297 respondents were recruited from 360 poultry farmers approached, a response rate of 82.5%. All respondents completed the face-to-face interview based on the questionnaire without missing data. These 297 chicken farmers fed between 300 and 25,000 chickens (median = 4000) each. Policy changes in Jiangsu Province encourage large-scale poultry farming while discouraging small-scale backyard poultry husbandry in order to increase the management standards of rural poultry farming. Of the respondents, 76.1% were male, while 50.8% and 30.6% were aged 46–55 years and ≥56 years, respectively; 76.1% of the respondents attained junior high school or lower educational achievement and over half (56.6%) had raised chickens for at least 10 years (Table 1).

**Table 1:** Respondents’ characteristics (*N* = 297)

Characteristics	*N*	%
Gender		
Female	71	23.9%
Male	226	76.1%
Age		
≦45 years	55	18.5%
46-55 years	151	50.8%
> 55 years	91	30.6%
Education		
primary or below	73	24.6%
Junior high school	153	51.5%
Senior high school or above	71	23.9%
Years raising poultry		
≦10 years	129	43.4%
10- 20 years	123	41.4%
> 20 years	45	15.2%

### A/H7N9 risk perceptions, intention to adopt and actual adoption of PPBs against A/H7N9

Respondents generally reported low perceived Vulnerability to A/H7N9 (mean value = 2.32 possible range 1–7) while perceived Severity of A/H7N9 was high (mean value = 5.96 possible range 1–7) (Additional file 2: Table S1). Perceived Self-efficacy was also high (mean value = 5.75 possible range 1–7) while perceived Response Efficacy (mean value = 4.81 possible range 1–7) and intention to adopt PPBs against A/H7N9 (mean value = 4.91 possible range 1–7) were moderate (Additional file 2: Table S1).

**Fig. 3:**
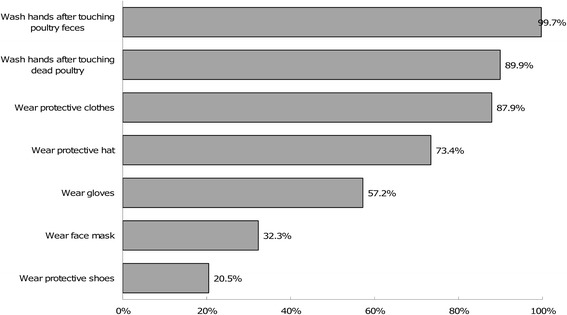
Actual adoption of personal protective behaviours against A/H7N9 among the respondents

Actual adherence to recommendations for washing hands after touching poultry feces (99.7%, 296/297), washing hands after touching dead poultry (89.9%, 267/297) and wearing protective clothing during poultry husbandry (87.9%, 261/297) were highly prevalent (Fig. 3). Only one third of respondents (32.3%, 96/297) wore face masks during routine husbandry practices and 20.5% (61/297) wore protective shoes. Around 12.5% (37/297) of the respondents adopted all the seven recommended protective behaviours. We ran a multivariate logistic model to regress adoption of all seven recommended protective behaviours on age, gender, educational attainment and years of raising poultry. The results showed that after adjustment for other demographics, respondents who had higher educational attainment were more likely to adopt all the seven recommended protective behaviours (Reference group: Primary or below; OR = 10.06, 95%CI: 2.08-48.62 for junior high school; OR = 9.53, 95%CI: 1.68-54.04 for senior high school or above), while respondents who had raised poultry for 10–20 years were less likely to adopt all the seven recommended protective behaviours (OR = 0.04, 95%CI: 0.01-0.20) relative to respondents who had raised poultry for less than 10 years.

### Relationships among demographics, PMT constructs and intention to adopt PPBs

**Fig. 4:**
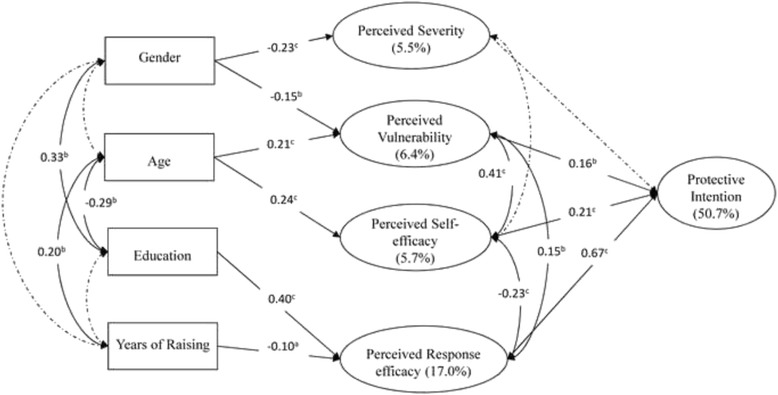
The results of structural equation model for understanding determinants on intention to adopt protective behaviours against A/H7N9 based on Protection Motivation Theory. ^a^ *p*<,0.05, ^b^ *p* < 0.01, ^c^ *p* < 0.001. The numbers on the paths are standardized path coefficient; the dotted line indicates the effect is not statistically significant

The hypothesized model was initially run with all paths from demographic variables to PMT constructs freely estimated (Model I). Then all insignificant paths (*p* ≥ 0.05) from the demographics to the PMT constructs were removed and the revised model (Model II) was re-run. Compared with Model I, Model II had comparable model fit indices, and the Satorra-Benter scaled chi-square difference test suggests that it fits to the data as well as Model I (Table 2). However, investigation of the residual matrix identified significant residual covariance between perceived Vulnerability and perceived Self-efficacy, between perceived Vulnerability and perceived Response Efficacy, between perceived Self-efficacy and perceived Severity, and between perceived Self-efficacy and perceived Response Efficacy. It indicates that the residual covariance of these latent variables is correlated. Therefore, the covariance between these variables was added to the model. The re-specified model (Model III) fits the data significantly better than Model II (Table 2). Investigation of the residual matrix revealed no significant residual covariance between the variables of Model III. Therefore, Model III was determined to be the optimal model. Compared with Model II, the parameters estimated for the structural part of Model III only differ slightly. The correlations between the four demographics age, gender, educational attainment and years of raising poultry were not higher than 0.33 (spearman correlation between age and gender), suggesting that multicollinearity is not a significant problem of the model [47]. The standardized covariance, path coefficients and the explained variance of each endogenous variable for Model III are shown in Fig. 4.

**Table 2:** Comparison of model fit indices of Model I, Model II and Model III

Nested models	χ^2^ (df)	Scaling correction factor	CFI	TLI	RMSEA (90% CI)	χ^2^ difference test (*p*)
Model I	495.03 (148)	1.12	0.94	0.93	0.09 (0.08-0.10)	-
Model II	499.87 (158)	1.12	0.94	0.93	0.08 (0.08-0.09)	*p*>0.10
Model III	420.69 (154)	1.13	0.96	0.95	0.08 (0.07-0.08)	*p*<0.001

Model II is nested within Model I and Model III

Compared with Model I, Model II removed the paths from gender to Perceived Self-efficacy and Perceived Response efficacy, from Age to Perceived Severity and Perceived Response efficacy, from Education to Perceived Severity, Perceived Vulnerability and Perceived Self-efficacy, and from years of raising poultry to Perceived Severity, Perceived Vulnerability and Perceived Response efficacy

Compared with Model II, Mode III added covariance for the relationships of Perceived Vulnerability with Perceived Self-efficacy and Perceived Response efficacy, and the relationships of Perceived Self-efficacy with Perceived Severity and Perceived Response efficacy

Gender was significantly associated with perceived Severity (β = −0.23) and perceived Vulnerability (β = -0.15), with female respondents perceiving higher Severity of, and Vulnerability to A/H7N9 infection than did males (Fig. 4). Age was significantly and positively associated with both perceived Vulnerability to A/H7N9 (β = 0.21) and perceived Self-efficacy (β = 0.24) in controlling A/H7N9. Educational attainment was only significantly and positively associated with perceived Response Efficacy (β = 0.40) while years of raising poultry were negatively associated with perceived Response Efficacy (β = -0.10). Subsequently, perceived Vulnerability to A/H7N9 (β = 0.16), perceived Self-efficacy (β = 0.21) and Response Efficacy (β = 0.67) were positively associated with Intention to adopt protective behaviours against A/H7N9. However, perceived Severity of A/H7N9 was not significantly associated with protective Intention, which is inconsistent with PMT predictions. The model explained 50.7% of the variance in Intention to adopt PPBs but only explained 5.5%, 6.4%, 5.7% and 17.0% Perceived Severity, Perceived Vulnerability, Perceived Self-efficacy and Perceived Response Efficacy, respectively.

The unstandardized direct effects of risk perceptions, and indirect effects of demographics via risk perceptions on Intention to adopt PPBs including the point estimate and 95% Bootstrapping confidence interval are shown in Table 3. Perceived Response Efficacy had strongest effect on behavioural Intention (point estimate = 0.54, 95%CI: 0.47-0.63). While education (point estimate = 0.35, 95%CI: 0.27-0.45) and age (point estimate = 0.11, 95%CI: 0.05-0.18) had significant positive indirect effects, gender and years of raising poultry did not had significant indirect effects on behavioural Intention (Table 3).

**Table 3:** The direct effects of risk perceptions and indirect effects of demographics on Intention to adopt personal protective behaviours via risk perceptions

Effects by exogenous variables	Point estimate (SE)	Bootstrapping (95%CI)	
		Lower	Upper
Direct effects			
Perceived Severity→Intention	-0.03 (0.03)	-0.08	0.03
Perceived Vulnerability→Intention	0.11 (0.04)^b^	0.04	0.18
Perceived Self-efficacy→Intention	0.40 (0.09)^c^	0.23	0.58
Perceived Response Efficacy→Intention	0.54 (0.04)^c^	0.47	0.63
Indirect effects			
Gender→Intention			
Via Perceived Severity	0.02 (0.02)	-0.02	0.06
Via Perceived Vulnerability	-0.05^a^	-0.11	−0.01
Total	-0.03 (0.03)	-0.10	0.02
Age→Intention			
Via Perceived Vulnerability	0.04 (0.02)^a^	0.01	0.09
Via Perceived Self-efficacy	0.07 (0.02)^b^	0.03	0.12
Total	0.11 (0.03)^c^	0.05	0.18
Education→Intention			
Via Perceived Response Efficacy	0.35 (0.04)^c^	0.27	0.45
Years of raising→Intention			
Via Perceived Response Efficacy	-0.08 (0.04)	-0.17	-0.00

^a^ *p* < 0.05, ^b^ *p* < 0.01, ^c^ *p* < 0.001; SE: Standard Error

### Information trust and the moderated effects of demographics on the relationships between information trust and PMT constructs

As shown in Table 4, over 99% (275/297) of respondents indicated mostly or completely trusting information from formal sources (TV, radio or newspaper). In contrast, only 14.5% (43/297) of respondents reported mostly or completely trusting informal information (information from friends or relatives). Male respondents and those with higher educational achievement were more likely to trust in formal or informal information compared with their counterparts (Table 4). Information trust did not differ by age and years of raising poultry. The universally high level of trust in formal information complicates testing for the moderated mediation models due to almost zero data variability. Therefore, the analysis only focused on the moderated effects of demographics on the relationships of TII with behavioural Intention via risk perceptions of A/H7N9.

**Table 4:** Trust in formal and informal information by demographic characteristics

Demographic characteristics	Trust in formal information		Trust in informal information	
	Trust (mostly/completely trustworthy)	*P*-value^a^	Trust (mostly/completely trustworthy)	*P*-value^b^
Gender				
Female	(69/71) 97.2%	0.011	(4/71) 5.6%	0.015
Male	(226/226)100%		(39/226) 17.3%	
Age				
≦45 years	(55/55) 100%	0.804	(4/55) 7.3%	0.241
46-55 years	(149/151) 98.7%		(24/151) 15.9%	
≧56 years	(91/91) 100%		(15/91) 16.5%	
Education				
Primary or below	(71/73) 97.3%	0.043	(7/73) 9.6%	<0.001
Junior high school	(153/153) 100%		(14/153) 9.2%	
Senior high school or above	(71/71) 100%		(22/71) 31.0%	
Years raising poultry				
≦10 years	(127/129) 98.4%	0.153	(12/129) 9.3%	0.080
≦20 years	(123/123) 100%		(22/123) 17.9%	
≧21 years	(45/45) 100%		(9/45) 20.0%	

^a^Fisher Exact test

^b^Pearson chi-square

The simple mediation model which hypothesized that effect of TII on Intention to adopt PPBs was mediated by perceived Severity, perceived Vulnerability, perceived Self-efficacy and perceived Response Efficacy were first tested. The simple mediation model fit well to the data (CFI=0.97, TLI=0.97, RMSEA=0.08 (90%CI: 0.07-0.09)). The results (Table 5) showed that only the indirect effects of TII on Intention through perceived Response Efficacy was significant (point estimate=0.57, 95%CI: 0.39-0.77).

**Table 5:** The direct and indirect effects of trust in informal information on Intention to adopt personal protective behaviours based on the simple mediation model

	Point estimate (SE)	Bootstrapping (95%CI)	
		Lower	Upper
Direct effect	-0.04 (0.13)	-0.28	0.24
Indirect effect			
Via perceived Severity	-0.01 (0.01)	-0.04	0.01
Via perceived Susceptibility	0.01 (0.02)	-0.02	0.07
Via perceived Self-efficacy	-0.07 (0.04)	-0.17	-0.00
Via perceived Response Efficacy	0.57 (0.10)^c^	0.39	0.77
Total indirect effect	0.50 (0.09)^c^	0.33	0.69
Total effect	0.46 (0.16)^b^	0.15	0.75

^b^ *p*<0.01, ^c^ *p*<0.001

Then multiple group modelling with each moderator being treated as a grouping variable was conducted to calculate and compare the indirect effects of TII on Intention via risk perceptions of A/H7N9 (Table 6). It shows that the indirect effects of TII on Intention via perceived Severity and Vulnerability were not significant across stratum of gender, age group and educational achievement. The indirect effect of TII on Intention via perceived Self-efficacy was only significant for female (point estimate=-0.24, 95%CI: -0.52–-0.06) and younger farmers (point estimate=-0.16, 95%CI: -0.30–-0.07). Age significantly moderated the mediation of TII with Intention via perceived Self-efficacy, with younger farmers who had more trust in informal information perceived lower self-efficacy. The indirect effects of TII on Intention via perceived Response Efficacy were significant across stratum of gender, age group and educational achievement excepting for framers who were older than 55 years. Age significantly moderated the mediation of TII with Intention via perceived Response Efficacy, with younger farmers who had more trust in informal information perceived higher response efficacy.

**Table 6:** The estimated conditional indirect effects of trust in informal information on intention to adopt personal protective behaviours against influenza A/H7N9 via risk perceptions

Moderator	Level	Conditional indirect effects of TII on Intention (Bootstrapping 95% CI) via:			
		Perceived Severity	Perceived Vulnerability	Perceived Self-efficacy	Perceived Response efficacy
Gender	Female	0.04 (-0.03, 0.13)	-0.16 (-0.39, 0.10)	-0.24 (-0.52, -0.06)^a^	0.30 (0.16-0.49)^b^
	Male	-0.02 (-0.08, 0.00)	0.05 (0.00, 0.15)	-0.05 (-0.17, 0.00)	0.56 (0.33, 0.80)^c^
Age group (years)	≦55	-0.01 (-0.04, 0.01)	0.02 (-0.02, 0.07)	**-0.16 (-0.30, -0.07)** ^**b**^	**0.77 (0.53, 1.00)** ^**c**^
	>55	-0.02 (-0.14, 0.04)	-0.03 (-0.21, 0.11)	0.05 (-0.03, 0.19)	0.19 (-0.10, 0.44)
Education	Junior middle or below	-0.02 (-0.08, 0.01)	-0.01 (-0.09, 0.06)	0.08 (-0.01, 0.26)	0.25 (0.01, 0.41)^a^
	Senior high or above	-0.00 (-0.07, 0.05)	0.08 (0.01, 0.29)	0.04 (-0.16, 0.27)	0.54 (0.23, 0.91)^b^

^a^ *p*<0.05, ^b^ *p*<0.01, ^c^ *p*<0.001; SE: Standard Error

The bold values indicate that effects were significant different across stratum of a moderator

## Discussion

Based on PMT, our study investigated how cognitive processes mediated the effects of demographics on motivation to adopt protective behaviours against A/H7N9, and how information trust interacted with demographics to influence A/H7N9 protection among the Chinese poultry farmers.

Generally, the study found that the respondents perceived A/H7N9 infection to be severe but did not perceive themselves to be vulnerable to the infection. This is consistent with one previous study conducted in The Netherlands which found that over 90% of the respondents perceived that avian influenza was a serious disease (mean score = 4.57, scale 1–5) but only 0.7% of them perceived themselves to be highly vulnerable to avian influenza (mean score = 1.69, scale 1–5) [48]. Chinese poultry farmers report more familiarity with poultry disease risk than do urbanites and are more optimistic about avoiding avian influenzas [12, 13]. Both familiarity and optimistic bias probably further account for the low perceived Vulnerability observed among these Jiangsu poultry farmers.

All PMT constructs were positively associated with PPB intention except for perceived Severity of A/H7N9 which was not significantly associated with PPB intention. The meta-analysis on the efficacy of PMT also indicates that the effect size of perceived Severity on protection motivation was the smallest among the four PMT constructs [32]. Given the small-to-moderate effect size of perceived Severity on behavioral intention, our small sample size may not be able to detect a significant association. However, while a previous review indicated that Self-efficacy had the strongest effect on behavioral intention [32], our study found that perceived Response Efficacy had the strongest effect on PPB intention, accounting for nearly 50% of the explained variance in PPB intention. For these poultry farmers, perceived Self-efficacy to adopt the preventive measures was generally high possibly because the recommended preventive measures are simple and thereby easily adopted. In this case, whether the preventive measures are believed to be effective or not to reduce risk of A/H7N9 plays a dominant role in determining their motivation to adopt the measures.

The finding that respondents with higher educational achievement had better compliance to PPBs is consistent with a previous study reporting better educated poultry traders were more likely to adopt PPBs when working [49]. Our study adds to the literature about the potential mechanism of how education influence adoption of PPBs. As indicated by the SEM, better educated respondents perceived higher response efficacy to prevent A/H7N9 which in turn was associated with higher intention to adopt protective behaviours against A/H7N9. Compared with other demographics, education had stronger indirect effects on intention to adopt protective behaviours via Perceived Response Efficacy. This suggests that interventions to promote belief in the efficacy of available protective behaviours among the less educated farmers may play a crucial role to improve compliance to self-protection against A/H7N9.

Consistent with our hypotheses, our study also found that females perceived higher personal Vulnerability to A/H7N9 and higher Severity of A/H7N9 compared with males. This finding may elucidate why compliance to recommended protection was usually higher among females during epidemics found in many descriptive studies [14]. However, the associations between age and PMT constructs were not consistent with our hypotheses. According to the SEM, older respondents perceived higher Vulnerability to A/H7N9 and higher Self-efficacy to prevent against A/H7N9 which in turn was significantly associated with higher intention to adopt PPBs. However, our study did not find a significant association between age and compliance to the recommended protective measures. This suggests factors other than these four PMT constructs may hinder translating the intention of adopting PPBs into actual behaviours among the older respondents. For example, perceived costs from taking the recommended behaviours (e.g., effort, time) may be greater among older people (e.g. older people need to take greater effort to alter their long-term habit) [32].

Our initial hypotheses that years of working with poultry could be associated with lower perceived Vulnerability and perceived Severity but higher perceived Self-efficacy and Response Efficacy were not supported. Years of working with poultry were only marginally associated with perceived Response Efficacy but the effect size was small. One possible reason could be that our measure of years of working with poultry may not be a good indicator for farmer’s experience with raising poultry. While a previous study did not find significant association between years of working with poultry and adoption of protective behaviours [11], our study found that respondents who had raised poultry for 10–20 years were less likely to adopt all the recommended protective measures than those who had raised poultry for less than 10 years after adjusting for age and educational attainment. Further studies are needed to explore the relationship between experience with poultry and adoption of PPBs among poultry farmers.

The indirect effect of TII on Intention to adopt PPBs was only significant via perceived Response Efficacy, with greater TII being associated with greater perceived Response Efficacy which was positively associated with behavioural Intention. This is not consistent with one previous study that TII was independent of efficacy belief but was positively associated with disease worry [30]. A possible reason for the inconsistent results could be that the former study was conducted among general public [30] while the current study was conducted among poultry farmers. Different types of informal information are probably communicated among different populations. Farmers may simply know what kinds of protective behaviours are available for preventing A/H7N9 through listening to their peer farmers and observing what they do. Age significantly moderated the mediation relationships of TII with behavioural Intention through perceived Self-efficacy and Response Efficacy. For younger farmers, more trust in information from peers was associated with perceived lower Self-efficacy but higher Response Efficacy, while corresponding associations were not statistically significant for older farmers. Such findings provide some insights about the possible types of information shared among younger poultry farmers. Younger poultry farmers who are usually better educated may be more aware of the effectiveness of available preventive measures in reducing risk of A/H7N9 but they may lack of confidence in adopting these preventive measures due to lack of skills in routine husbandry practices with protective measures. For example, they may find it more inconvenient to wear gloves or protective clothes in their routine husbandry practice. Therefore, it may be important to provide training for improving young poultry farmers’ skills of taking protective measures.

This study has several limitations. First, the cross-sectional design excluded causal inference. Second, without follow-up data, this study cannot examine the gap between intention and subsequent adoption of PPBs, though current adherence to PPBs was measured, and past behaviour is the best predictor for future behaviours [50]. Third, actual PPBs were dichotomously (yes/no) measured and because of social desirability bias, may provide less accurate assessment of actual compliance. Furthermore, while multilevel SEM (MSEM) may be more appropriate for our data that were collected based on sampling stratified by clusters, MSEM cannot be conducted due to lack of data on clusters. Therefore, our single-level SEM assuming that subjects were independent within clusters may underestimate the sampling variance, which may result in inflation of the type I error [45].

## Conclusions

Jiangsu poultry farmers generally perceived A/H7N9 Severity as high, but personal Vulnerability to infection as low, these variables being weakly associated with intention to adopt PPBs, possibly due to perceived personally-irrelevant risk. The moderate perceived Response Efficacy of respondents and its strongest association with PPB intention reflect that interventions designed to enhance perceived Response Efficacy may effectively motivate adoption of PPBs among these poultry farmers. Education appears to influence intention to adopt PPBs through its positive association with Response Efficacy, suggesting that Response Efficacy should be promoted among lower educated farmers. For example, information about how and why a recommended behavior can eliminate or decrease risk of infection should be clearly presented and framed in an easily-understood way for lower educated framers. The study also adds to the literature that gender appears to influence on intention to adopt PPBs through its effects on perceived disease Vulnerability and Severity, while age may influence on behavioural intention through its effects on perceived disease Vulnerability and perceived Self-efficacy in prevention. Greater TII was associated with higher Intention to take protective measures through its positive association with perceived Response Efficacy. Age significantly moderated the association between TII and perceived Self-efficacy, and between TII and perceived Response Efficacy, with younger farmers who had greater TII perceived lower Self-efficacy but higher Response Efficacy. Young poultry farmers may just simply obtain the information about the availability of effective preventive measures against A/H7N9 from listening to what their peer farmers say and observing what they do. This suggests that interventions utilizing farmer peers to communicate and train poultry farmers in taking protective measures during routine husbandry practice may be effective to promote adoption of PPBs among poultry farmers.

## Additional files

Additional file 1: Figure S1. Map of Jiangsu Province showing the sampling sites. Note: Maps of China and Jiangsu Province were reproduced based on maps provided by WIKIPEDIA available from https://en.wikipedia.org/wiki/Jiangsu (TIFF 1511 kb).

Additional file 2: Table S1. and **S2.** The measuring items for the constructs of Protection Motivation Theory and descriptive statistics (DOCX 25 kb).

Additional file 3: Data for analysis (XLS 163 kb).

## Abbreviations

AVE: Average variance extracted
PMT: Protection Motivation Theory
PPBs: Personal protective behaviours
SEM: Structural equation modeling
TII: Trust in Informal Information
