# Supplementary figures and images for: Avian influenza A/H7N9 risk perception, information trust and adoption of protective behaviours among poultry farmers in Jiangsu Province, China
Source: BMC Public Health. 2017 May 18;17:463. doi: 10.1186/s12889-017-4364-y (PMC5437685; doi:10.1186/s12889-017-4364-y)

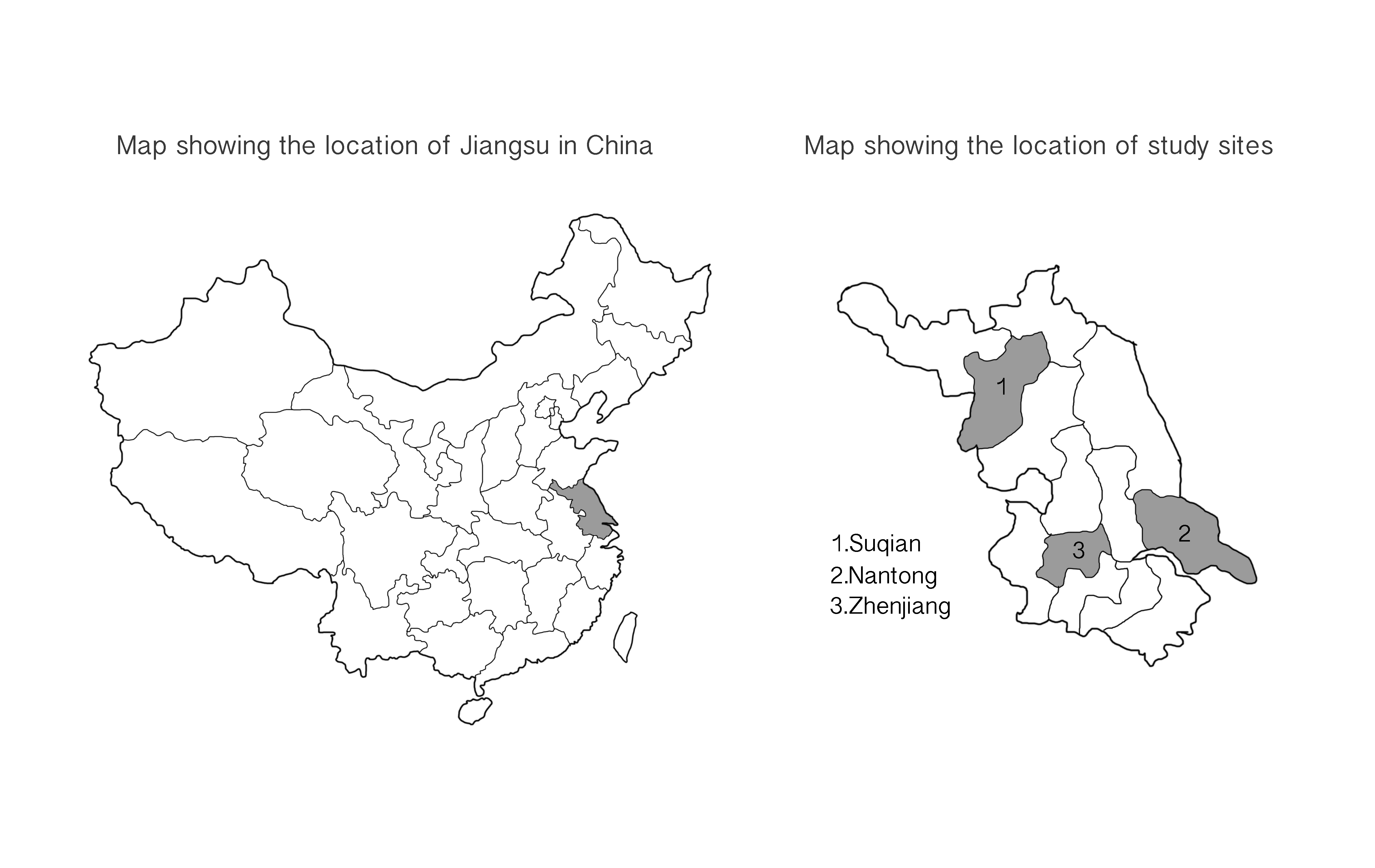

Supplement: Supplementary file 1 — Map of Jiangsu Province showing the sampling sites. Note: Maps of China and Jiangsu Province were reproduced based on maps provided by WIKIPEDIA available from https://en.wikipedia.org/wiki/Jiangsu (TIFF 1511 kb). [file 12889_2017_4364_MOESM1_ESM.tiff]
