# Supplementary material for: Avian influenza A/H7N9 risk perception, information trust and adoption of protective behaviours among poultry farmers in Jiangsu Province, China
Source: BMC Public Health. 2017 May 18;17:463. doi: 10.1186/s12889-017-4364-y (PMC5437685; doi:10.1186/s12889-017-4364-y)
Supplement: Supplementary file 2 — and S2. The measuring items for the constructs of Protection Motivation Theory and descriptive statistics (DOCX 25 kb). [file 12889_2017_4364_MOESM2_ESM.docx]

Appendix Table 1 The measuring items for the constructs of Protection Motivation Theory and descriptive statistics

| Variables | Items | Mean | SD | | Response  scales |
| --- | --- | --- | --- | --- | --- |
| **Perceived Severity** |  |  |  |  | |
| SEV1 | It is a very serious matter if human infected the H7N9 virus. | 6.00 | 1.37 | | 1–7 agreement |
| SEV2 | Human infected with H7N9 will be more serious than being infected with other influenzas. | 5.99 | 1.41 | |  |
| SEV3 | It is hard to cure if human infected with H7N9 virus. | 5.87 | 1.41 | |  |
| SEV4 | It is very dangerous if human infected with H7N9 virus. | 5.99 | 1.41 | |  |
| **Perceived Vulnerability** |  |  |  |  | |
| VUL1 | I may be infected withH7N9 virus if I do not take self-protective measures. | 2.45 | 1.31 | | 1–7 agreement |
| VUL2 | Other chicken farmers may be infected with H7N9 virus if they do not take self-protective measures. | 2.39 | 1.30 | |  |
| VUL3 | It is possible to be infected with H7N9 virus if someone engages in poultry feeding for long time. | 2.11 | 1.35 | |  |
| **Perceived Self-Efficacy** |  |  |  |  | |
| SEL1 | I can do it if I want to take self-protective measures to prevent against H7N9 infection. | 5.71 | 0.58 | | 1–7 agreement |
| SEL2 | I can make a decision myself about whether to take self-protective measures or not. | 5.78 | 0.58 | |  |
| SEL3 | It is not a difficult thing for me to take self-protective measures to prevent against H7N9 infection. | 5.77 | 0.64 | |  |
| **Perceived Response Efficacy** |  |  |  |  | |
| RES1 | The self-protective measures can reduce the risk of contracting H7N9 infection. | 4.79 | 1.13 | | 1–7 agreement |
| RES2 | The self-protective measures can protect me against H7N9 infection. | 4.80 | 1.21 | |  |
| RES3 | The self-protective measures are effective for preventing against H7N9 infection. | 4.83 | 1.15 | |  |
| **Protective Intention** |  |  |  |  | |
| INT1 | I intend to take self-protective measures during periods of H7N9 epidemic. | 4.93 | 0.92 | | 1–7 agreement |
| INT2 | I am certain to take self-protective measures during periods of H7N9 epidemic | 4.89 | 0.93 | |  |
| INT3 | I will consider taking self-protective measures during periods of H7N9 epidemic. | 4.91 | 0.93 | |  |
| Actual Protective Behaviours |  |  |  |  | |
| BE1 | Do you wear gloves in your routine husbandry practices? | 0.57 | 0.50 | | Yes=1  No=0 |
| BE2 | Do you wear protective clothes in your routine husbandry practices? | 0.88 | 0.47 | |  |
| BE3 | Do you wear a face mask in your routine husbandry practices? | 0.32 | 0.47 | |  |
| BE4 | Do you wear a protective hat in your routine husbandry practices? | 0.73 | 0.44 | |  |
| BE5 | Do you wear protective shoes in your routine husbandry practices? | 0.21 | 0.40 | |  |
| BE6 | Do you wash hands after touching the dead poultry in your routine husbandry practices? | 0.90 | 0.30 | |  |
| BE7 | Do you wash hands after touching the poultry feces in your routine husbandry practices? | 1.00 | 0.06 | |  |

SD: standard deviation

Appendix Table 2 The reliability and validity assessment of the constructs

|  | 1 | 2 | 3 | 4 | 5 | Cronbach’s α | AVE | Skewness | Kurtosis |
| --- | --- | --- | --- | --- | --- | --- | --- | --- | --- |
| 1.Perceived Severity | 0.99 | — | — | — | — | 0.99 | 0.97 | －0.97 | -0.56 |
| 2.Perceived Vulnerability | 0.04 | 0.97 | — | — | — | 0.96 | 0.94 | 1.03 | 0.48 |
| 3.Self-Efficacy | 0.00 | 0.43 | 0.92 | — | — | 0.90 | 0.84 | 0.12 | -0.01 |
| 4.Response Efficacy | -0.07 | -0.05 | -0.02 | 0.99 | — | 0.98 | 0.97 | -0.24 | -0.44 |
| 5.Protective Intention | -0.07 | 0.21 | 0.26 | 0.65 | 0.980 | 0.97 | 0.96 | 0.03 | -0.29 |

Appendix Table 3 Normalized residual matrix of the variables in Model III

|  | 1 | 2 | 3 | 4 | 5 | 6 | 7 | 8 | 9 | 10 | 11 | 12 | 13 | 14 | 15 | 16 | 17 | 18 | 19 | 20 |
| --- | --- | --- | --- | --- | --- | --- | --- | --- | --- | --- | --- | --- | --- | --- | --- | --- | --- | --- | --- | --- |
| 1.INT1 | 0.11 |  |  |  |  |  |  |  |  |  |  |  |  |  |  |  |  |  |  |  |
| 2.INT2 | 0.12 | 0.10 |  |  |  |  |  |  |  |  |  |  |  |  |  |  |  |  |  |  |
| 3.INT3 | 0.10 | 0.09 | 0.10 |  |  |  |  |  |  |  |  |  |  |  |  |  |  |  |  |  |
| 4.SEV1 | -0.55 | -0.94 | 0.28 | -0.01 |  |  |  |  |  |  |  |  |  |  |  |  |  |  |  |  |
| 5.SEV2 | -1.12 | -1.27 | 0.18 | -0.01 | -0.01 |  |  |  |  |  |  |  |  |  |  |  |  |  |  |  |
| 6. SEV3 | -0.35 | -0.51 | 0.66 | -0.10 | 0.02 | -0.01 |  |  |  |  |  |  |  |  |  |  |  |  |  |  |
| 7. SEV4 | -0.71 | -1.14 | 0.44 | -0.01 | -0.02 | 0.02 | -0.01 |  |  |  |  |  |  |  |  |  |  |  |  |  |
| 8. VUL1 | 0.09 | 0.09 | -0.03 | 0.85 | 0.33 | 0.89 | 0.70 | 0.02 |  |  |  |  |  |  |  |  |  |  |  |  |
| 9. VUL2 | 0.14 | 0.15 | 0.04 | 0.40 | -0.11 | 0.38 | 0.16 | 0.02 | 0.02 |  |  |  |  |  |  |  |  |  |  |  |
| 10. VUL3 | 0.51 | 0.60 | 0.46 | -0.57 | -0.95 | -0.79 | -0.89 | 0.00 | 0.02 | 0.01 |  |  |  |  |  |  |  |  |  |  |
| 11. SEL1 | 0.13 | 1.53 | 0.67 | 0.12 | 0.25 | 1.66 | 0.17 | 0.91 | 0.56 | 0.03 | -0.04 |  |  |  |  |  |  |  |  |  |
| 12. SEL2 | -1.37 | -0.40 | -0.54 | 0.60 | 0.68 | 1.07 | 0.36 | -0.52 | -0.71 | -0.57 | -0.15 | -0.04 |  |  |  |  |  |  |  |  |
| 13. SEL3 | 0.48 | 1.37 | 0.66 | 0.00 | 0.04 | 0.95 | 0.11 | 0.32 | -0.20 | -0.39 | -0.08 | 0.05 | -0.04 |  |  |  |  |  |  |  |
| 14. RES1 | 0.29 | -0.31 | 0.43 | -1.25 | -1.36 | -1.26 | -1.09 | 0.06 | 0.22 | 1.79 | 0.55 | -0.55 | 1.01 | -0.03 |  |  |  |  |  |  |
| 15. RES2 | 0.34 | -0.01 | 0.79 | -1.66 | -1.74 | -1.65 | -1.59 | -0.61 | -0.35 | 1.55 | 0.37 | -1.00 | -0.03 | -0.04 | -0.03 |  |  |  |  |  |
| 16. RES3 | -0.17 | -0.72 | 0.10 | -1.06 | -1.18 | -1.14 | -0.94 | 0.38 | 0.62 | 2.41 | 0.39 | -0.89 | 0.62 | -0.02 | -0.04 | -0.03 |  |  |  |  |
| 17. Gender | -0.35 | 0.69 | -0.61 | -0.08 | 0.36 | -0.58 | -0.02 | 0.19 | 0.13 | 0.80 | 1.17 | -0.04 | 0.00 | 0.36 | 0.31 | -0.08 | 0.00 |  |  |  |
| 18. Age | 0.09 | 0.74 | -0.03 | 0.10 | 0.50 | 0.60 | 0.39 | -0.03 | 0.38 | -0.71 | 1.12 | -0.39 | -0.81 | 1.50 | 0.91 | 0.93 | 0.00 | 0.00 |  |  |
| 19. Education | 1.17 | 1.85 | 1.66 | -1.43 | -1.09 | -1.03 | -1.46 | -0.09 | 0.09 | 0.68 | -0.36 | 0.38 | -0.26 | -0.11 | 0.57 | -0.22 | 0.00 | 0.00 | 0.00 |  |
| 20. Year of raising | 0.54 | -0.03 | -0.97 | 0.62 | -0.02 | -1.23 | 0.24 | 0.03 | 0.25 | -0.47 | 1.05 | -0.35 | -0.78 | 0.42 | -0.45 | -0.18 | 0.00 | 0.00 | 0.00 | 0.00 |
